# Reductions in hospitalisations and emergency department visits with early antibiotic initiation in nontuberculous mycobacterial lung disease

**DOI:** 10.1183/23120541.00963-2023

**Published:** 2024-07-22

**Authors:** Kevin Winthrop, Catherine Waweru, Mariam Hassan, Sara Burns, Matthew Lucci, Anjan Chatterjee

**Affiliations:** 1 Oregon Health and Science University, Portland State University School of Public Health, Portland, OR, USA; 2 Insmed Incorporated, Bridgewater Township, NJ, USA; 3 Panalgo, Boston, MA, USA

## Abstract

**Background:**

While antibiotics are recommended for treatment of nontuberculous mycobacterial lung disease (NTMLD), the impact of early antibiotic initiation on healthcare resource utilisation is unclear. This study compared healthcare resource utilisation with early *versus* delayed antibiotic initiation in NTMLD.

**Methods:**

A retrospective, claims database study (Merative MarketScan) of patients diagnosed with NTMLD between 1 July 2015 and 30 June 2019. Patients were divided into early antibiotic initiation, *i.e.* ≤3 months after the first medical claim for NTMLD (index date), and delayed antibiotic initiation groups. Hospitalisations and outpatient visits during a 2-year post-index period were compared to baseline per treatment group; a difference-in-difference analysis compared early and delayed antibiotic initiation groups adjusting for confounding.

**Results:**

Out of 481 NTMLD treated patients, 364 (76%) and 117 (24%) comprised the early and delayed antibiotic initiation groups, respectively. The early antibiotic initiation group showed significant reductions from baseline in hospitalisations (all-cause, respiratory) and emergency department (ED) visits at follow-up. A significant increase from baseline in mean number of hospitalisations per patient was observed in the delayed antibiotic initiation group in year 1 post-index. Compared to delayed antibiotic initiation, the early antibiotic initiation group showed significantly greater reductions in all-cause hospitalisations in years 1 and 2 post-index (relative risk 0.62 (95% CI 0.41–0.95) and 0.62 (95% CI 0.39–0.98), respectively), and in respiratory-related hospitalisations.

**Conclusions:**

The early antibiotic initiation group showed significant reductions from baseline in hospitalisations and ED visits over time. Compared to delayed antibiotic initiation, early antibiotic initiation was associated with significantly greater reductions in hospitalisations.

## Shareable abstract

**Patients with nontuberculous mycobacterial lung disease initiating early antibiotic treatment showed significant reductions in hospitalisations and emergency department visits over a 2-year follow-up period** https://bit.ly/3xe5ZKJ

## Introduction

Nontuberculous mycobacterial lung disease (NTMLD) is a rare, chronic, progressive lung infection caused by the mycobacterial group of pathogens, of which *Mycobacterium avium* complex (MAC) is the most common [1–3]. Environmental factors such as proximity to large areas of water, and host factors such as older age, female sex and presence of underlying pulmonary comorbidities (*e.g.* COPD and bronchiectasis), are known risk factors of NTMLD [3, 4].

Patients with NTMLD commonly present with cough, which is often productive (*i.e.* coughing up phlegm, mucus or blood) [5–7]. Other symptoms include fatigue, fever, haemoptysis, shortness of breath, malaise and night sweats [6]. NTMLD negatively impacts patient health-related quality of life (HRQoL) and has been associated with an increased mortality risk [8, 9]. In addition, NTMLD is associated with increased rates of healthcare resource utilisation (HCRU) in the form of all-cause hospitalisations, respiratory-related hospitalisations and emergency department (ED) visits [10, 11].

The current American Thoracic Society (ATS)/European Respiratory Society (ERS)/European Society of Clinical Microbiology and Infectious Diseases (ESCMID)/Infectious Disease Society of America (IDSA) guidelines [1] recommend that combination antibiotic treatment should be initiated in patients diagnosed with NTMLD, especially in the context of positive acid-fast bacilli sputum smears and lung cavitary disease. For patients with MAC lung disease, an antibiotic regimen is recommended consisting of a macrolide (clarithromycin or azithromycin), ethambutol and rifampin, with the addition of parenteral amikacin in cases of cavitary disease or inhaled liposomal amikacin for treatment-refractory disease. The same macrolide-based antibiotic regimen is also an option in the treatment of NTMLD caused by *M. kansasii* and *M. xenopi*. For *M. abscessus*, recommended therapy consists of drugs found susceptible by *in vitro* susceptibility testing and can include amikacin, cefoxitin, tigecycline, omadacycline, imipenem, macrolides, moxifloxacin, linezolid, clofazimine or bedaquiline [1].

Culture conversion is the main goal of treatment in NTMLD [1]. Patients who achieve culture conversion should receive an additional 12 months of continuous antibiotic treatment from the date of their first negative sputum culture. While antibiotic treatment can be lengthy and complex [1], it can mitigate symptoms [12] and disease progression [13] and improve patient HRQoL [14]. Nevertheless, treatment guidelines acknowledge that a “watchful waiting” approach may be preferred in some instances, after taking into consideration the pathogenicity of the organism, risks and benefits of therapy, the patient's willingness to adhere to therapy, and treatment goals [1].

While antibiotics are the mainstay of treatment in NTMLD, the impact on HCRU of initiating early antibiotic treatment is unclear. The objective of our study was to compare hospitalisations and outpatient visits in patients who initiated antibiotic treatment early after NTMLD diagnosis with patients who delayed antibiotic initiation.

## Methods

### Data source

This was a retrospective, observational cohort study based on claims data from the Merative MarketScan Commercial Claims and Encounters database. The MarketScan databases are administrative claims databases that contain deidentified data on inpatient and outpatient claims, outpatient prescription claims, clinical utilisation records and healthcare expenditures of ∼40 million United States residents who receive employer-sponsored health insurance [15, 16]. As a claims database study, no patients were enrolled and no human tissue samples were collected or used. Because the study did not involve the collection, use or transmittal of individually identifiable data, institutional review board approval was not required and the study was considered exempt according to 45CFR46.101(b) (4): Existing Data and Specimens – No Identifiers.

### Study cohort

**FIGURE 1:**
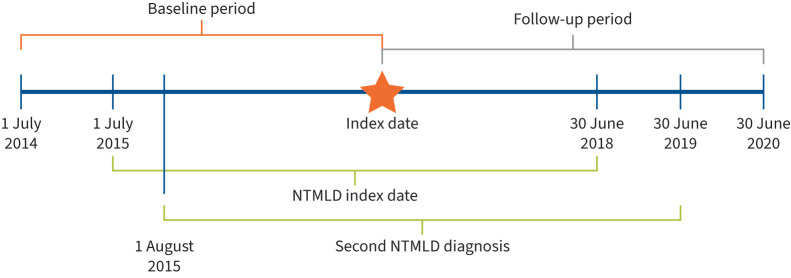
Study design. NTMLD: nontuberculous mycobacterial lung disease.

Patients with two or more NTMLD medical claims with International Classification of Diseases, Ninth Revision, Clinical Modification (ICD-9-CM) code 031.0 or tenth revision (ICD-10-CM) code A31.0 between 1 July 2014 and 30 June 2020 were identified from the database (figure 1). Included patients must have had a first NTMLD medical claim (index date) between 1 July 2015 and 30 June 2018, with a second medical claim ≥30 days but no more than 12 months after the index date (figure 1) [17]. Other eligibility criteria included continuous enrolment (*i.e.* no gaps in coverage >90 days) beginning 12 months prior to index date (referred to hereafter as the baseline period) and continuing for ≥24 months post-index date, age >18 years at index date, and initiation of antibiotic treatment for NTMLD after the index date. Patients with a diagnosis of tuberculosis (TB) were excluded to avoid potential disease misclassification because of similarities in antibiotic regimens used to treat TB and NTMLD.

### Treatment definitions

Treatment of NTMLD was defined as receiving antibiotics from at least two drug classes (table 1), each lasting ≥28 days and within 30 days of each other. As no standard definition of early or delayed antibiotic initiation was identified in the published literature, the mean time from index date to first NTMLD treatment initiation was used to stratify the NTMLD cohort into early and delayed antibiotic initiation groups. The early antibiotic initiation group was defined as patients whose time to treatment initiation from index date was ≤3 months, while >3 months post-index date defined the delayed antibiotic initiation group.

**TABLE 1:** Antibiotic drug classes used to treat nontuberculous mycobacterial lung disease

Drug class	Drug name
**Macrolide**	Azithromycin, clarithromycin
**Ethambutol**	Ethambutol
**Rifamycin**	Rifampin, rifabutin
**Aminoglycoside**	Amikacin, streptomycin
**Fluoroquinolone**	Ciprofloxacin, moxifloxacin, levofloxacin
**Carbapenem**	Imipenem, meropenem
**Oxazolidinone**	Linezolid, tedizolid
**Glycylcycline**	Tigecycline
**Cephalosporin antibiotic**	Cefoxitin
**Tetracycline**	Omadacycline
**Diarylquinoline**	Bedaquiline
**Lipophilic riminophenazine**	Clofazimine

### Study outcomes

The primary outcomes of interest were all-cause hospitalisations, respiratory-related hospitalisations, and outpatient ED and non-ED visits. All-cause hospitalisation was defined as hospitalisation for any reason. Respiratory-related hospitalisation was defined as a hospitalisation with a principal claim for a respiratory disease (ICD-9-CM 460–519 or ICD-10-CM J00–J99). Non-ED visits included outpatient clinic visits and procedural visits, *e.g.* radiology, laboratory, injections and spirometry. The time points considered for all outcomes were the 12 months prior to the index date (baseline period) and years 1 and 2 after the index date.

### Statistical analysis

Baseline patient demographic and clinical characteristics were summarised using descriptive statistics. Comparisons of patient baseline characteristics between early and delayed antibiotic initiation groups were conducted using Chi-squared tests for categorical variables, and t-tests for continuous variables. Treatment outcomes described were the proportions of patients with hospitalisations and outpatient visits at baseline, year 1 and year 2 post-index period. The rates of hospitalisations and outpatient visits were described as mean±sd per patient. A pre–post analysis was performed, in which all-cause hospitalisations, respiratory disease-related hospitalisations, ED and non-ED visits in year 1 and 2 post-index date were compared with the baseline period per treatment group. To account for the nonindependence of this pre–post analysis, McNemar Chi-squared tests were used to compare the proportions, while Wilcoxon signed rank tests were used to compare rates. Furthermore, a sensitivity analysis examined trends in hospitalisations and outpatient visits with shorter *versus* longer delays in antibiotic initiation in the delayed antibiotic initiation group.

A difference-in-difference (DID) analysis was used to assess the impact on HCRU of early *versus* delayed antibiotic initiation over the 2-year post-index period, adjusting for patient characteristics. Multivariate generalised linear models with binomial distributions (for categorical outcomes) and Poisson distribution (for counts) were built, that included treatment group (early *versus* delayed), follow-up period (year 1, year 2 post-index period *versus* baseline), and an interaction term of treatment group and follow-up period, to evaluate the significance of the DID interaction term. A relative risk <1 in the interaction term indicated that the early antibiotic initiation group showed a greater reduction in HCRU than the delayed antibiotic initiation group during the follow-up period. The adjusted analyses all retained age at index date, gender, baseline Charlson Comorbidity Index, baseline COPD, baseline bronchiectasis and baseline immunosuppressive use as model covariates. Other baseline comorbidities and symptoms were retained if their p-values were ≤0.15 in the final multivariate model [18]. Baseline comorbidities and symptoms were identified using ICD-9 and ICD-10 codes from inpatient and outpatient medical claims (supplementary table S1).

All analyses were performed using the Instant Health Data software (Panalgo, Boston, MA, USA) and R (version 3.2.1; R Foundation for Statistical Computing, Vienna, Austria). p-values <0.05 were considered statistically significant.

## Results

### NTMLD treatment groups

**FIGURE 2:**
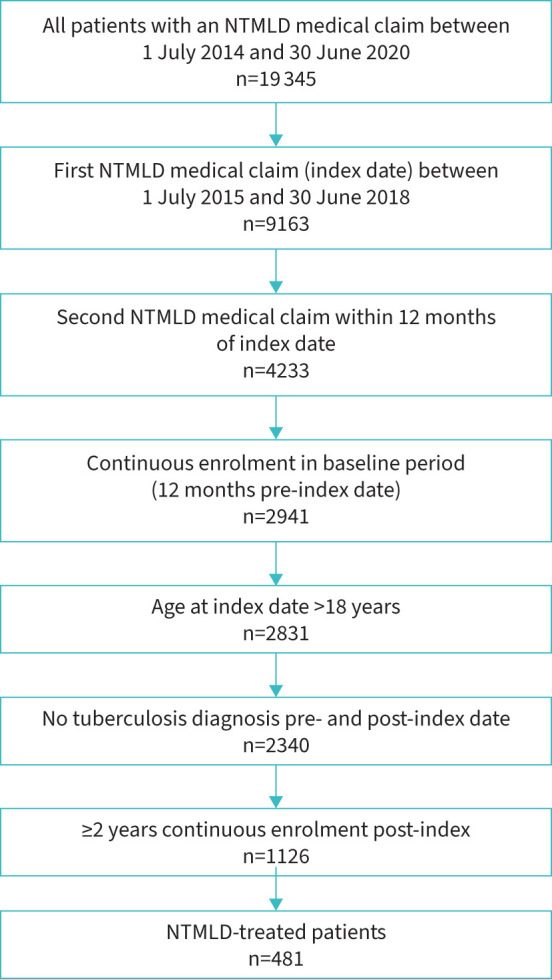
Flow diagram showing eligibility criteria. NTMLD: nontuberculous mycobacterial lung disease.

After applying the eligibility criteria, 481 patients fulfilled the definition of NTMLD treated (figure 2). Of these, 364 (75.7%) and 117 (24.3%) patients comprised the early and delayed antibiotic initiation groups, respectively. Patients in the early antibiotic initiation group were significantly younger than those in the delayed antibiotic initiation group (mean 58.9 *versus* 62.0 years, p=0.03) and had a lower proportion of females (57.7% *versus* 78.6%, p<0.0001) (table 2). Other baseline characteristics were balanced between the two groups.

**TABLE 2:** Characteristics of nontuberculous mycobacterial lung disease (NTMLD) treated patients by antibiotic initiation group

	Early antibiotic initiation^#^	Delayed antibiotic initiation^¶^	p-value^+^
**Patients**	364 (75.7)	117 (24.3)	
**Age at index date^§^ years**	58.9±12.8	62.0±13.6	0.03
**Age ≥65 years**	97 (26.7)	46 (39.3)	0.01
**Female**	210 (57.7)	92 (78.6)	<0.0001
**Baseline CCI^ƒ^**	1.85±1.87	1.97±1.93	0.56
**Baseline immunosuppressant use^ƒ,##^**	187 (51.4)	63 (53.9)	0.72
**Baseline pulmonary comorbidities and symptoms^ƒ^**			
Cough	219 (60.2)	74 (63.3)	0.63
Dyspnoea	167 (45.9)	50 (42.7)	0.63
Pneumonia	143 (39.3)	45 (38.5)	0.96
COPD	134 (36.8)	44 (37.6)	0.96
Bronchiectasis	118 (32.4)	48 (41.0)	0.11
Asthma	91 (25.0)	38 (32.5)	0.14
Fatigue	77 (21.2)	20 (17.1)	0.41
Emphysema	45 (12.4)	19 (16.2)	0.36
Haemoptysis	32 (8.8)	14 (12.0)	0.40
Idiopathic interstitial lung disease	42 (11.5)	11 (9.4)	0.64
Lung cancer	15 (4.1)	6 (5.1)	0.84
Idiopathic pulmonary fibrosis	6 (1.7)	1 (0.9)	Not estimable
Lung transplant	5 (1.4)	0 (0)	Not estimable
**Baseline nonpulmonary comorbidities^ƒ^**			
Hypertension	152 (41.8)	51 (43.6)	0.81
GORD	93 (25.6)	36 (30.8)	0.32
Other cancers	56 (15.4)	16 (13.7)	0.76
Cardiovascular	62 (17.0)	21 (18.0)	0.93
Smoking	50 (13.7)	12 (10.3)	0.41
Diabetes	44 (12.1)	19 (16.2)	0.32
Malnutrition	25 (6.9)	14 (12.0)	0.12
Obesity	31 (8.5)	7 (6.0)	0.49

Data are presented as n (%) or mean±sd, unless otherwise stated. CCI: Charlson Comorbidity Index; GORD: gastro-oesophageal reflux disease. ^#^: time to treatment initiation from index date ≤3 months; ^¶^: time to treatment initiation from index date >3 months; ^+^: Chi-squared test used to compare categorical variables, t-test used to compare continuous variables; ^§^: defined as the date of the first NTMLD diagnosis; ^ƒ^: 12 months prior to index date; ^##:^ immunosuppressant use included the following: methotrexate, leflunomide, sulfasalazine, tacrolimus, cyclosporine, cyclophosphamide, azathioprine, infliximab, etanercept, golimumab, certolizumab, adalimumab, tofacitinib, baricitinib, upadacitinib, rituximab, abatacept, ixekizumab, secukinumab, corticosteroids (oral and inhaled), anakinra, brodalumab, ustekinumab.

A total of 43 (37%) patients in the delayed antibiotic initiation group initiated antibiotic treatment >3 to ≤6 months post-index date; 41 (35%) patients initiated >6 to ≤12 months post-index date; and 33 (28%) patients initiated treatment >12 months post-index date. A majority of the NTMLD-treated cohort (n=445, 92.5%) was initiated on a macrolide-based regimen (supplementary table S2).

### Healthcare resource utilisation

#### Hospitalisations

**FIGURE 3:**
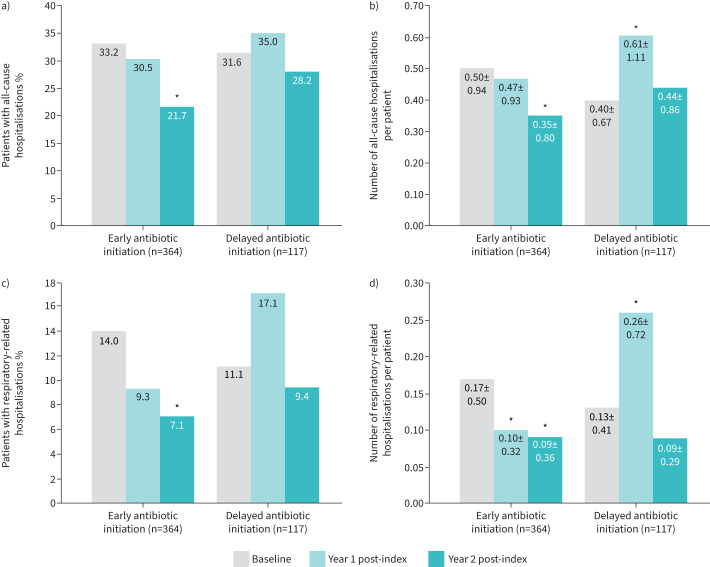
All-cause and respiratory-related hospitalisations from baseline to 2 years post-index per antibiotic initiation group. a) Proportion of patients with all-cause hospitalisations; b) mean±sd number of all-cause hospitalisations per patient; c) proportion of patients with respiratory-related hospitalisations; d) mean±sd number of respiratory-related hospitalisations per patient. McNemar Chi-squared test was used to compare proportions of hospitalisations at year 1 and year 2 *versus* baseline (a, c). Wilcoxon signed rank test was used to compare mean number of hospitalisations at year 1 and year 2 *versus* baseline (b, d). *: p<0.05.

The early antibiotic initiation group showed a significant decrease from baseline in the proportion and mean±sd number of all-cause hospitalisations per patient (21.7% *versus* 33.2% p=0.0002 and 0.35±0.80 *versus* 0.50±0.94; p=0.003, respectively) at year 2 post-index (figure 3a and b)*.* No significant differences from baseline were observed in the proportions of patients with all-cause hospitalisations in years 1 and 2 in the delayed antibiotic initiation group. The delayed antibiotic initiation group saw a significant increase in the mean±sd number of all-cause hospitalisations per patient (0.61±1.11 *versus* 0.40±0.67, p=0.03) in year 1 post-index from baseline, but no significant differences from baseline in year 2.

Similar trends were observed with respiratory-related hospitalisations. The early antibiotic initiation group showed a significant decrease in the proportion of patients with respiratory-related hospitalisations from baseline in year 2 (7.1% *versus* 14.0%, p*=*0.002) (figure 3c). Significant reductions in mean±sd numbers of respiratory-related hospitalisations per patient from baseline were observed in the early antibiotic initiation group in years 1 and 2 post-index (year 1 0.10±0.32 *versus* 0.17±0.50, p=0.02; year 2 0.09±0.36 *versus* 0.17±0.50, p=0.007) (figure 3d). No significant differences from baseline were observed in the proportion of patients with respiratory-related hospitalisations in year 1 and 2 in the delayed antibiotic initiation group. A significant increase in the mean±sd number of respiratory-related hospitalisations was observed in the delayed antibiotic initiation group in year 1 (0.26±0.72 *versus* 0.13±0.41, p=0.03), but there were no significant differences from baseline in year 2. Respiratory-related hospitalisations during the post-index period were most commonly associated with pneumonia (28%) and COPD (24%) (supplementary table S3).

#### Outpatient visits

**FIGURE 4:**
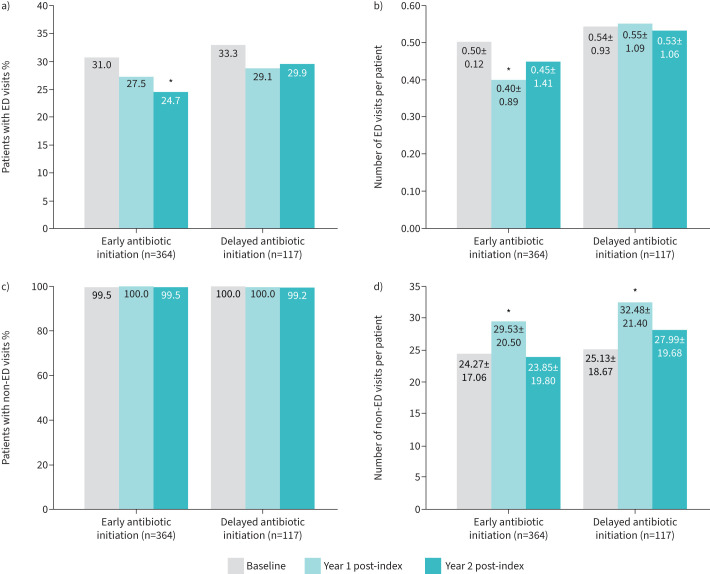
Emergency department (ED) and non-ED visits from baseline to 2 years post-index per antibiotic initiation group. a) Proportion of patients with ED visits; b) mean±sd number of ED visits per patient; c) proportion of patients with non-ED visits; d) mean±sd number of ED visits per patient. Index date: date of first nontuberculous mycobacterial lung disease diagnosis. McNemar Chi-squared test was used to compare proportions of ED visits at year 1 and year 2 *versus* baseline (a, c). Wilcoxon signed rank test was used to compare mean number of ED visits at year 1 and year 2 *versus* baseline (b, d). *: p<0.05.

The early antibiotic initiation group showed a significant reduction from baseline in the proportion of patients with ED visits (24.7% *versus* 31.0%, p=0.04*)* in year 2; and in mean±sd number of ED visits per patient in year 1 post-index (0.40±0.89 *versus* 0.50±1.12, p=0.04) (figure 4a and b). No significant differences in the proportions of non-ED visits were identified in either group during the post-index period (figure 4c). However, both treatment groups showed mean rates of non-ED visits ranging between 24.3 and 32.5 visits per patient per year between baseline and year 2 of follow-up (figure 4d). Both treatment groups had a significant increase from baseline in mean±sd number of non-ED visits per patient in year 1 (early antibiotic initiation group 29.53±20.50 *versus* 24.27±17.06; delayed antibiotic initiation group 32.48±21.40 *versus* 25.13±18.67; all p<0.0001), but no significant differences from baseline in year 2.

#### *Shorter* versus *longer delay in antibiotic initiation*

Using a cut-off of 6 months, the delayed antibiotic initiation group was stratified into shorter delay (*i.e.* >3 to ≤6 months from index date) and longer delay (>6 months from index date) subgroups (supplementary figure S1). Baseline proportions and mean rates of all-cause and respiratory-related hospitalisations, and ED visits, were higher in the shorter delay than longer delay subgroups. In addition, both subgroups showed numerical, albeit nonsignificant increases from baseline in the rates of all-cause and respiratory-related hospitalisations in year 1, with a substantial increase from baseline in the rate of all-cause hospitalisations observed in the shorter delay subgroup (0.56±0.73 *versus* 0.84±1.25). Patients in the shorter delay subgroup (n=43) showed significant reductions from baseline in the proportion and mean±sd number of respiratory-related hospitalisations per patient in year 2 (18.6% *versus* 2.3%, p=0.04 and 0.23±0.57 *versus* 0.02±0.15, p=0.02, respectively).

#### DID analysis

Results of the DID analysis are provided in table 3. Compared to the delayed antibiotic initiation group, the early antibiotic initiation group showed significantly greater reductions in the rates of all-cause hospitalisations in year 1 (adjusted relative risk 0.62, 95% CI 0.41–0.95; p=0.03) and year 2 (adjusted relative risk 0.62, 95% CI 0.39–0.98; p=0.04) post-index. In addition, the early antibiotic initiation group showed significantly greater reductions in the proportion and rate of respiratory-related hospitalisations in year 1 (adjusted relative risk 0.31, 95% CI 0.12–0.83; p=0.02 and adjusted relative risk 0.29, 95% CI 0.14–0.60; p<0.001, respectively) compared to the delayed antibiotic initiation group.

**TABLE 3:** Difference-in-difference analysis of healthcare resource utilisation with early *versus* delayed antibiotic initiation for nontuberculous mycobacterial lung disease (NTMLD)

	Difference-in-difference interaction term (unadjusted, adjusted^#^)			
	Year 1 relative risk (95% CI)	p-value	Year 2 relative risk (95% CI)	p-value
**Proportions**				
All-cause hospitalisations				
Unadjusted	0.76 (0.40–1.41)	0.38	0.66 (0.34–1.26)	0.20
Adjusted	0.71 (0.36–1.42)	0.34	0.61 (0.30–1.24)	0.17
Respiratory-related hospitalisations				
Unadjusted	0.38 (0.16–0.93)	0.03	0.57 (0.21–1.52)	0.26
Adjusted	0.31 (0.12–0.83)	0.02	0.52 (0.18–1.52)	0.23
ED visits				
Unadjusted	1.03 (0.54–1.95)	0.94	0.85 (0.45–1.62)	0.63
Adjusted	1.03 (0.52–2.04)	0.93	0.84 (0.43–1.66)	0.62
**Rates**				
All-cause hospitalisations				
Unadjusted	0.62 (0.41–0.95)	0.03	0.62 (0.39–0.98)	0.04
Adjusted	0.62 (0.41–0.95)	0.03	0.62 (0.39–0.98)	0.04
Respiratory-related hospitalisations				
Unadjusted	0.29 (0.14–0.60)	<0.001	0.71 (0.29–1.73)	0.46
Adjusted	0.29 (0.14–0.60)	<0.001	0.71 (0.29–1.73)	0.46
ED visits				
Unadjusted	0.78 (0.52–1.18)	0.24	0.93 (0.62–1.39)	0.71
Adjusted	0.78 (0.52–1.18)	0.24	0.93 (0.62–1.39)	0.71

The difference-in-difference analysis examined whether changes from baseline in healthcare resource utilisation at year 1 and year 2 post-index period were significantly different between early *versus* delayed antibiotic treatment initiation groups. ED: emergency department. ^#^: regression models all adjusted for age at index date, gender, baseline Charlson Comorbidity Index, baseline COPD, baseline bronchiectasis, baseline immunosuppressive therapy. Other baseline comorbidities and symptoms were included if their p-values were <0.15 in the final multivariate model.

## Discussion

In our study of NTMLD treated patients, early antibiotic initiation was associated with significant reductions in HCRU over time. Patients in the early antibiotic initiation group, *i.e.* initiated treatment within 3 months of an initial medical claim for NTMLD, saw significant reductions from baseline in the proportions and mean±sd numbers of all-cause and respiratory-related hospitalisations, as well as ED visits during the 2-year post-index period. The delayed antibiotic initiation group saw a significant increase from baseline in the rates of all-cause and respiratory-related hospitalisations in year 1, with no significant improvements from baseline observed in year 2. A DID analysis found that after adjusting for patient demographic and clinical characteristics, early antibiotic initiation was associated with reductions in all-cause and respiratory-related hospitalisations compared to delayed antibiotic initiation; with significantly lower reductions in all-cause hospitalisations observed in years 1 and 2 post-index, and in respiratory-related hospitalisations in year 1. These results highlight the need for timely discussions between patients diagnosed with NTMLD and their healthcare providers of the potential benefits of initiating early antibiotic treatment.

Considering that the majority (63%) of patients in the delayed antibiotic initiation group started treatment >6 months after NTMLD diagnosis, the increased rates of hospitalisations observed in year 1 post-index may have resulted from progressive lung disease prior to initiating treatment. Several studies have described increasing lung damage associated with failure to initiate antibiotic treatment for NTMLD [12, 13, 19–22]. Watanabe *et al*. [22] reported that patients untreated for MAC lung disease experienced a worsening of radiographic factors, including nodules, infiltration, cavities and ectasis. While antibiotic treatment was shown to improve nodular and infiltration factors, lung cavitation and ectasis proved to be irreversible after treatment initiation, leading the authors to conclude that earlier antibiotic initiation could mitigate the accumulation of irreversible factors that lead to lung damage [22]. Our study found that the significant increase in hospitalisations observed in the delayed antibiotic initiation group in year 1 did not extend into year 2 of the follow-up period. Our findings support the observations by Watanabe *et al.* [22], that trends towards worsening outcomes in patients who delay antibiotic initiation may be reversed once treatment is initiated.

A sensitivity analysis found that patients with shorter delays in antibiotic initiation had higher baseline rates of all-cause and respiratory-related hospitalisations than patients with longer delays, including a larger increase from baseline in all-cause hospitalisations in year 1 post-index. It is possible that patients in the shorter delay subgroup experienced more rapid disease progression than longer delay patients, resulting in higher rates of hospitalisations at baseline and year 1. Notably, both shorter and longer delay subgroups saw increases in the rates of all-cause and respiratory-related hospitalisations in year 1, suggesting that any delay in antibiotic treatment initiation could increase hospitalisations in patients with NTMLD. The results of the sensitivity analysis should be interpreted with caution given small patient numbers in each subgroup.

Both early and delayed antibiotic treatment initiation groups showed high numbers of non-ED visits per year, with significant increases from baseline in year 1. There is evidence that USA patients with NTMLD tend to have a high number of outpatient visits [2]. Increased non-ED visits may be due to monitoring of treatment outcomes (*i.e.* culture conversion, clinical symptoms), adverse events management and monitoring for disease progression.

The delayed antibiotic initiation group had a higher proportion of patients who were older and female compared with the early antibiotic initiation group. This observation is concerning, given that older age and female gender tend to be predisposing factors for NTMLD [3]. The decision to delay antibiotic treatment initiation in NTMLD may be due to various factors including presence of mild disease, concerns around treatment side-effects, drug–drug interactions, pill burden and patient preference [3, 23]. Furthermore, some patients experience spontaneous culture conversion in the absence of antibiotic treatment [1, 24]. Nonetheless, the conundrum faced by patients with NTMLD and physicians during treatment decision making is in predicting who might be at risk of disease progression if antibiotic treatment is not initiated. It is thus important for healthcare providers to understand patients’ concerns around treatment for NTMLD, educate patients who may choose to delay antibiotic initiation on the potential risks of delaying treatment, and ensure regular monitoring in patients who elect to delay antibiotic initiation in order to encourage timely antibiotic initiation upon disease progression.

We identified two other studies that examined the association of time to antibiotic initiation with outcomes in NTMLD, although neither included HCRU. Im *et al*. [25] found no significant association between time to treatment initiation and culture conversion or death. Consistent with our findings, Kwak *et al*. [14] reported that untreated patients who eventually required treatment experienced worsening HRQoL prior to initiating antibiotic treatment, which improved once patients initiated treatment, regardless of microbiological response. Our study findings are in agreement with Im *et al*. [25] in that both early and delayed antibiotic initiation groups appeared to benefit from initiating antibiotic treatment for NTMLD. For the early antibiotic initiation, the benefit of treatment occurred by reducing proportions and rates of hospitalisations and ED visits at follow-up from the pre-diagnosis period. In the delayed antibiotic initiation group, the benefit of antibiotic initiation occurred by reversing trends towards increasing rates of hospitalisations in the following 2 years.

### Study strengths and limitations

Our study has several strengths. This is the only study, to our knowledge, to describe and compare HCRU with early *versus* delayed antibiotic treatment initiation for NTMLD. Our study used a validated approach of defining NTMLD in claims, reported to have a high positive predictive value (PPV) of 72% [17]. In order to avoid including patients without NTMLD in the study cohort, only patients who initiated antibiotic regimens consistent with NTMLD treatment guidelines were included. This approach has been shown to have a PPV of 100%, meaning that all patients in our study cohort can be considered as truly diagnosed with NTMLD [26]. In addition, a pre–post study design using each patient as their own control minimised bias by adjusting for time-invariant patient confounders [27]. Furthermore, a DID analysis compared changes in hospitalisations and ED visits over time between early and delayed antibiotic initiation groups, adjusting for some confounding factors [28].

Our study has a number of limitations. Our analysis utilised administrative claims that are used primarily for billing purposes, leading to a possibility of disease misclassification or miscoding. In addition, the MarketScan database uses claims obtained from USA-based patients who predominantly receive commercial insurance through large employers. Patients aged ≥65 years, Medicaid patients and patients insured through small and medium employers tend to be underrepresented [15]. Additionally, these results may not be generalisable to other countries. Another limitation inherent to all claims databases is that data on important disease-related factors such as virulence of the infecting species, presence of cavitary disease, culture conversion results and NTMLD symptom severity were lacking and could not be adjusted for in the DID analysis. These disease-related factors are important confounders that may influence treatment decision-making and impact HCRU, and our study results should therefore be interpreted in the context of these limitations. Furthermore, while our study utilised an algorithm with high PPV to identify patients with NTMLD in claims, this algorithm has been shown to have only 34% sensitivity in identifying all patients with NTMLD, both treated and untreated, meaning that we may have inadvertently excluded some patients with NTMLD who initiated antibiotic treatment [26]. Finally, it is possible that the first NTMLD diagnosis occurred prior to the date of the first medical claim for NTMLD, leading some patients to be misclassified in the early antibiotic initiation group as opposed to delayed.

### Conclusions

The early antibiotic initiation group showed significant reductions from baseline, in hospitalisations and ED visits over time. When compared to delayed antibiotic initiation, early antibiotic initiation was associated with significantly greater reductions in hospitalisations.

## Supplementary material

10.1183/23120541.00963-2023.Supp1 **Please note:** supplementary material is not edited by the Editorial Office, and is uploaded as it has been supplied by the author.
Supplementary material 00963-2023.SUPPLEMENT
