# Supplementary material for: Reductions in hospitalisations and emergency department visits with early antibiotic initiation in nontuberculous mycobacterial lung disease
Source: ERJ Open Res. 2024 Jul 22;10(4):00963-2023. doi: 10.1183/23120541.00963-2023 (PMC11261350; doi:10.1183/23120541.00963-2023)
Supplement: Supplementary file 1 [file 00963-2023.SUPPLEMENT.pdf]

Supplementary Material

SUPPLEMENTARY TABLE 1. Listing of *ICD-9-CM* and *ICD-10-CM* codes for baseline comorbidities and symptoms

**Clinical comorbidities**	***ICD-9-CM* code(s)**	***ICD-10-CM* code(s)**
**Pulmonary comorbidities and symptoms**		
Asthma	493	J45
Bronchiectasis	494	J47
COPD	491, 492, 496	J41-J44
Cough	786	R05
Dyspnea	518, 770, 786	R06
Emphysema	492	J43
Fatigue	780	R53
Hemoptysis	784,786	R04
Idiopathic interstitial lung disease	515-516	J84
Idiopathic pulmonary fibrosis	516.3	J84.1
Lung transplant	V42	Z94
Lung cancer	162	C34
Pneumonia	480-486	J12−J18
Smoking history	V15	Z87
**Non-pulmonary comorbidities**		
Cardiovascular disease	398, 402, 404, 414, 425, 428	I09, I11, I13, I25, I42-I43, I50.x, P29.0
Other cancers	140-161, 163-172, 174-176, 179-195, 200-208, 238	C00-C26, C30-C33, C37-C41, C43-C45, C4A, C58, C60-C75, C81-C85, C88, C90
Diabetes mellitus	250	E10−E11, E13
GERD	530	K21
Hypertension	401	I10−I13, I15
Malnutrition	260-269	E40- E46
Obesity	278	E66

COPD: chronic obstructive pulmonary disease; GERD: gastroesophageal reflux disease; *ICD-9/10-CM*: International Classification of Diseases, Ninth/Tenth Revision, Clinical Modification.

SUPPLEMENTARY TABLE 2. Initial antibiotic regimen received in the NTMLD-treatment cohort

Antibiotic regimen, n (%)	Patients receiving treatment (N=481)
Macrolide + ethambutol + rifamycin	269 (55.9)
Macrolide + ethambutol	56 (11.6)
Macrolide + rifamycin	41 (8.5)
Macrolide + fluoroquinolone	32 (6.7)
Macrolide + rifamycin + fluoroquinolone ± ethambutol	10 (2.1)
Macrolide + ethambutol + fluoroquinolone	6 (1.2)
Macrolide + other[a]	31 (6.4)
Nonmacrolide-containing regimen[b]	36 (7.5)

[a]Other: oxazolidinone (n=16); amikacin (n=12); diarylquinoline (n=1); oxazolidinone + amikacin (n=1); diarylquinoline + amikacin (n=1).

[b]Nonmacrolide-containing regimen: ethambutol + rifamycin (n=24); fluoroquinolone + oxazolidinone (n=5); fluoroquinolone + rifamycin (n=2); ethambutol + fluoroquinolone (n=2); ethambutol + fluoroquinolone + rifamycin (n=2); amikacin + oxazolidinone (n=1).

NTMLD: nontuberculous mycobacterial lung disease.

SUPPLEMENTARY TABLE 3. Respiratory conditions associated with respiratory-related hospitalisations based on *ICD-9-CM* and *ICD-10-CM* diagnosis codes.

	**N (%)**
**Total number of respiratory-related hospitalisations**	**110 (100)**
**Pneumonia**	**31 (28.2)**
**ICD9/ ICD10 Diagnosis Codes**	
J18.9 - Pneumonia, unspecified organism	7 (1.4)
J15.1 - Pneumonia due to Pseudomonas	5 (1.0)
J15.212 - Pneumonia due to Methicillin resistant Staphylococcus aureus	3 (0.6)
J15.9 - Unspecified bacterial pneumonia	3 (0.6)
J15.8 - Pneumonia due to other specified bacteria	2 (0.4)
J15.6 - Pneumonia due to other Gram-negative bacteria	2 (0.4)
J18.1 - Lobar pneumonia, unspecified organism	2 (0.4)
J13 - Pneumonia due to Streptococcus pneumoniae	1 (0.2)
J12.89 - Other viral pneumonia	1 (0.2)
J12.3 - Human metapneumovirus pneumonia	1 (0.2)
J15.211 - Pneumonia due to Methicillin susceptible Staphylococcus aureus	1 (0.2)
J15.5 - Pneumonia due to Escherichia coli	1 (0.2)
J11.08 - Influenza due to unidentified influenza virus with specified pneumonia	1 (0.2)
J11.00 - Influenza due to unidentified influenza virus with unspecified type of pneumonia	1 (0.2)
**Chronic obstructive pulmonary disease (COPD)**	**26 (23.6)**
**ICD9/ ICD10 Diagnosis Codes**	
J44.1 - Chronic obstructive pulmonary disease with (acute) exacerbation	18 (3.5)
J44.0 - Chronic obstructive pulmonary disease with (acute) lower respiratory infection	7 (1.4)
J44.9 - Chronic obstructive pulmonary disease, unspecified	1 (0.2)
**Respiratory Failure**	**11 (10.0)**
**ICD9/ ICD10 Diagnosis Codes**	
J96.01 - Acute respiratory failure with hypoxia	4 (0.8)
J96.21 - Acute and chronic respiratory failure with hypoxia	3 (0.6)

J96.00 - Acute respiratory failure, unspecified whether with hypoxia or hypercapnia	1 (0.2)
J96.22 - Acute and chronic respiratory failure with hypercapnia	3 (0.6)
**Bronchiectasis**	**11 (10.0)**
**ICD9/ ICD10 Diagnosis Codes**	
J47.1 - Bronchiectasis with (acute) exacerbation	6 (1.2)
J47.9 - Bronchiectasis, uncomplicated	2 (0.4)
J47.0 - Bronchiectasis with acute lower respiratory infection	1 (0.2)
494.0 - Bronchiectasis without acute exacerbation	1 (0.2)
494.1 - Bronchiectasis with acute exacerbation	1 (0.2)
**Pneumonitis**	**6 (5.5)**
**ICD9/ ICD10 Diagnosis Codes**	
J69.0 - Pneumonitis due to inhalation of food and vomit	6 (5.5)
**Asthma**	**4 (3.6)**
**ICD9/ ICD10 Diagnosis Codes**	
J45.41 - Moderate persistent asthma with (acute) exacerbation	3 (0.6)
J45.901 - Unspecified asthma with (acute) exacerbation	1 (0.2)
**Pulmonary fibrosis**	**4 (3.6)**
**ICD9/ ICD10 Diagnosis Codes**	
J84.10 - Pulmonary fibrosis, unspecified	4 (3.6)
**Other**	**17 (15.5)**
**ICD9/ ICD10 Diagnosis Codes**	
491.22 - Obstructive chronic bronchitis with acute bronchitis	1 (0.2)
J20.9 - Acute bronchitis, unspecified	1 (0.2)
J98.4 - Other disorders of lung	1 (0.2)
J95.830 - Postprocedural hemorrhage of a respiratory system organ or structure following a respiratory system procedure	1 (0.2)
J38.3 - Other diseases of vocal cords	1 (0.2)
J93.83 - Other pneumothorax	1 (0.2)
J01.90 - Acute sinusitis, unspecified	1 (0.2)
J84.112 - Idiopathic pulmonary fibrosis	1 (0.2)
J43.9 - Emphysema, unspecified	1 (0.2)
J84.89 - Other specified interstitial pulmonary diseases	1 (0.2)
J95.811 - Postprocedural pneumothorax	1 (0.2)
J85.3 - Abscess of mediastinum	1 (0.2)
J67.9 - Hypersensitivity pneumonitis due to unspecified organic	1 (0.2)

dust	
J86.0 - Pyothorax with fistula	1 (0.2)
J94.0 - Chylous effusion	1 (0.2)
J93.9 - Pneumothorax, unspecified	1 (0.2)
J98.2 - Interstitial emphysema	1 (0.2)

SUPPLEMENTARY FIGURE 1 Hospitalisations and ER visits from baseline to 2 years post-index among patients with shorter delays[a] *vs* longer delays[b] in antibiotic treatment initiation. a) Proportion of patients with all-cause hospitalisations, b) mean (SD) number of all-cause hospitalisations per patient. c) Proportion of patients with respiratory-related hospitalisations, and d) mean (SD) number of respiratory-related hospitalisations per patient. e) Proportion of patients with ER visits, and f) mean (SD) number of ER visits per patient. [a]Shorter delay: antibiotic treatment initiation >3 to ≤6 months from index date. [b]Longer delay: antibiotic treatment initiation >6 months from index date. *p<0.05. McNemar Chi-square test used to compare proportions of hospitalisations or ER visits at Year 1 and Year 2 *vs* baseline (a, c, e). Wilcoxon signed rank test used to compare mean number of hospitalisations or ER visits at Year 1 and Year 2 *vs* baseline (b, d, f). Index date: date of first NTMLD diagnosis. ER, emergency room; NTMLD, nontuberculous mycobacterial lung disease.

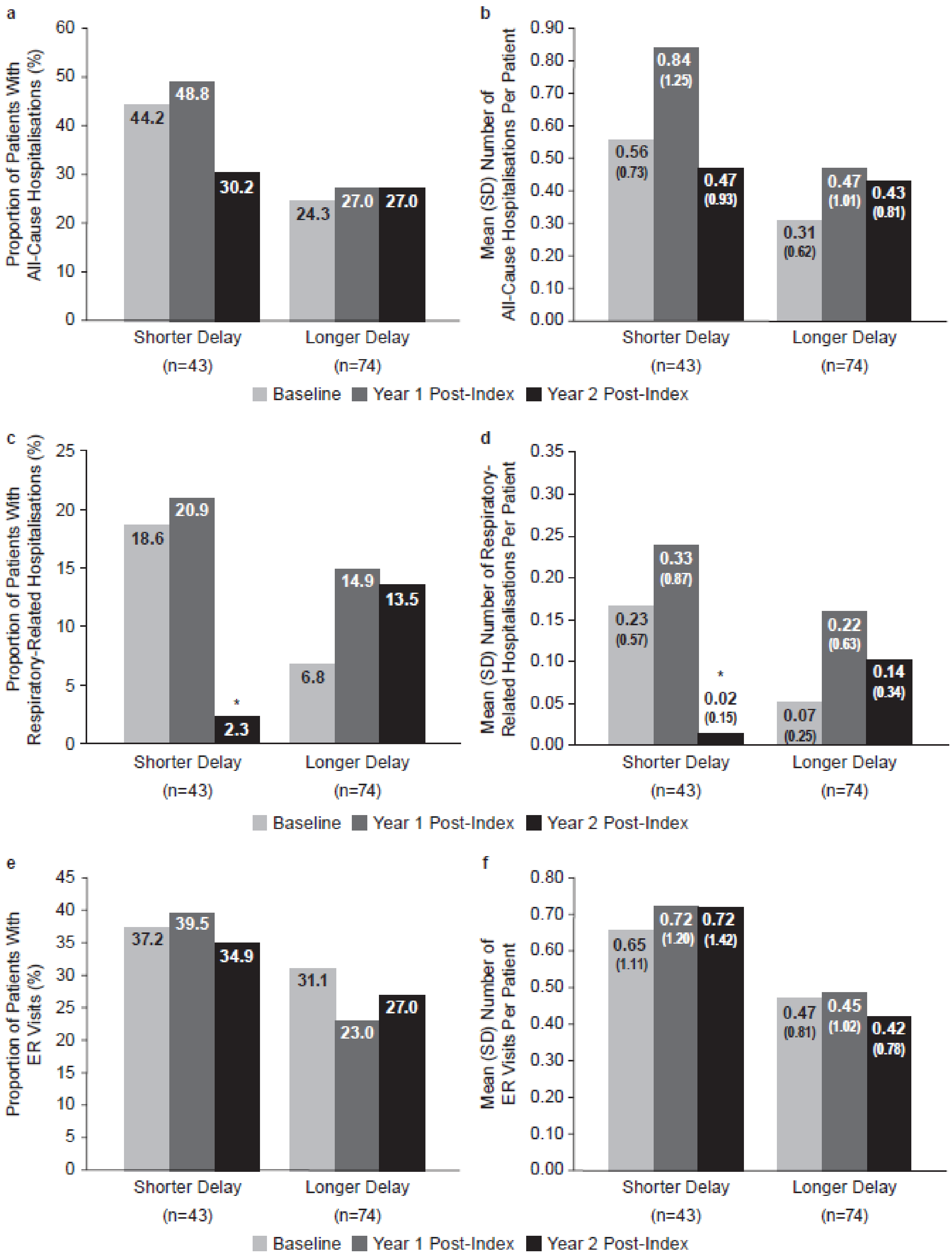
a
Proportion of Patients With All-Cause Hospitalisations (%)
0
10
20
30
40
50
60
44.2
48.8
30.2
24.3
27.0
27.0
Shorter Delay (n=43)
Longer Delay (n=74)
b
Mean (SD) Number of All-Cause Hospitalisations Per Patient
0.00
0.10
0.20
0.30
0.40
0.50
0.60
0.70
0.80
0.90
0.56 (0.73)
0.84 (1.25)
0.47 (0.93)
0.31 (0.62)
0.47 (1.01)
0.43 (0.81)
Shorter Delay (n=43)
Longer Delay (n=74)
Baseline
Year 1 Post-Index
Year 2 Post-Index
c
Proportion of Patients With Respiratory-Related Hospitalisations (%)
0
5
10
15
20
25
18.6
20.9
*
2.3
6.8
14.9
13.5
Shorter Delay (n=43)
Longer Delay (n=74)
d
Mean (SD) Number of Respiratory-Related Hospitalisations Per Patient
0.00
0.05
0.10
0.15
0.20
0.25
0.30
0.35
0.23 (0.57)
0.33 (0.87)
*
0.02 (0.15)
0.07 (0.25)
0.22 (0.63)
0.14 (0.34)
Shorter Delay (n=43)
Longer Delay (n=74)
Baseline
Year 1 Post-Index
Year 2 Post-Index
e
Proportion of Patients With ER Visits (%)
0
5
10
15
20
25
30
35
40
45
37.2
39.5
34.9
31.1
23.0
27.0
Shorter Delay (n=43)
Longer Delay (n=74)
f
Mean (SD) Number of ER Visits Per Patient
0.00
0.10
0.20
0.30
0.40
0.50
0.60
0.70
0.80
0.65 (1.11)
0.72 (1.20)
0.72 (1.42)
0.47 (0.81)
0.45 (1.02)
0.42 (0.78)
Shorter Delay (n=43)
Longer Delay (n=74)
Baseline
Year 1 Post-Index
Year 2 Post-Index
